# Relationship trust in individuals with multiple sclerosis: a network analysis approach

**DOI:** 10.1007/s13760-026-03130-9

**Published:** 2026-07-08

**Authors:** Cristiana Văcăraș, Larisa Cristina Ioana Tînc, Veronica Văcăraș, Răzvan Predatu, Cătălina Crișan, Dafin F. Mureșanu, Vitalie Văcăraș

**Affiliations:** 1 Faculty of Medicine, Iuliu Hațieganu University of Medicine and Pharmacy, Cluj-Napoca, Romania; 2 Doctoral School “Evidence-based assessment and psychological interventions”, Babeș- Bolyai University, Cluj-Napoca, Romania; 3 Department of Clinical Psychology and Psychotherapy, Babeș-Bolyai University, Cluj- Napoca, Romania; 4 International Institute for the Advanced Studies of Psychotherapy and Applied Mental Health, Babeș-Bolyai University, Cluj-Napoca, Romania; 5 Civil Professional Psychotherapy Society, Cluj-Napoca, Romania; 6 Department of Neurosciences, Faculty of Medicine, Iuliu Hațieganu University of Medicine and Pharmacy, Cluj-Napoca, Romania; 7 Department of Psychiatry, Cluj Emergency County Hospital, Cluj-Napoca, Romania; 8 Department of Neurology, Cluj Emergency County Hospital, Cluj-Napoca, Romania

**Keywords:** Multiple sclerosis, Chronic illness, Relationship trust, Network analysis, Caring

## Abstract

**Introduction:**

The aim of this study was to assess the pattern of mutual relations within the Relationship Trust Scale (RTS) in a sample of individuals diagnosed with multiple sclerosis.

**Methods:**

The participants comprised 189 individuals diagnosed with multiple sclerosis (M_age_ = 43.39 years; 123 females, 61 males, 5 missing). Analyses were conducted using RStudio and JASP.

**Results:**

The results indicated that, in terms of strength, the Cares node (Item: “My partner cares about my worries, feelings, joys, and troubles”) had the highest strength in the network. Furthermore, regarding expected influence, the analysis revealed that the Cares node had the highest expected influence in the network, followed by the Availability node (Item: "Whenever I am upset my partner is always available, supportive, and I feel like he/she’s there for me”), and Consideration in Decisions node (Item: “My partner takes me into account when making a decision.“). Also, the Cares node had the strongest associations with Consideration in Decisions, Safety, and Respect nodes. Network stability analyses indicated that interpretations should be focused primarily on expected influence (CS-coefficient = 0.286), which exceeded the minimum recommended threshold (Epskamp et al., 2018), while other centrality indices showed lower stability. Betweenness and closeness centrality were not interpreted, as these measures may be conceptually unsuitable for psychological networks (Bringmann et al., 2019). All findings should be considered exploratory and preliminary.

**Conclusion:**

These exploratory findings appear to highlight potentially relevant relationship processes among individuals diagnosed with multiple sclerosis that may be targeted to improve the overall quality of their relationships.

**Supplementary Information:**

The online version contains supplementary material available at https://doi.org/10.1007/s13760-026-03130-9.

## Introduction

Multiple Sclerosis (MS) is a progressive neurological disorder that compromises the protective layer surrounding nerves, called myelin, resulting in communication disruptions within the central nervous system [18]. This damage leads to symptoms such as fatigue, cognitive impairment, affective disorders, emotional burden, and diminished physical functioning. MS is a multifaceted condition influenced by various genetic and environmental factors, including vitamin D exposure, Epstein–Barr virus infection, obesity, and smoking [13]. It predominantly affects young adults and exhibits an increasing incidence and prevalence globally, although its exact etiology remains uncertain [13]. The worldwide prevalence of MS has risen to 2.8 million in 2020, with a global prevalence rate of 35.9 [95% CI: 35.87, 35.95] per 100,000 people [33]. This upward trend in MS prevalence is evident across all world regions since 2013 [33], with an estimated diagnosis rate of one person every 5 min, and women being twice as likely to develop MS compared to men [33].

The profound impact of MS on daily life significantly affects social and quality of life factors [1, 17, 18]. Individuals with MS experience disruptions in physical and emotional well-being, interpersonal relationships, and daily activities, accompanied by an increased risk of anxiety and depressive symptoms [1, 17].

One important social aspect affected by the presence of MS is the quality of intimate relationships. McPheters and Sandberg, [22] conducted a cross-sectional study involving 54 couples to explore the association among couple relationship quality, physical functioning, and depression in MS patients and their partners. The findings revealed significant correlations between both MS patients and partners’ ratings of couple relationship quality and the physical functioning of MS patients. The findings indicate that higer couple relationship quality was associated with better MS patient physical functioning and relate to fewer health problems. Although causality cannot be inferred due to the study’s cross-sectional design, results pointed out that there is a significant relationship between couple relationship quality and MS patient physical functioning. Moreover, couples with higher-quality relationships may offer better support to each other during challenging situations, such as living with MS.

Similarly, Wright and Kiropoulos [36] observed that the level of disability in people with multiple sclerosis was a significant negative predictor of intimate relationship quality. Furthermore, it was observed that factors such as insufficient marital adjustment and the risk of separation have a serious stressor effect on MS patients. Another cross-sectional study was conducted to examine the psychosocial situation of couples where one partner had MS, regarding anxiety, depression, and stress communication [8]. The findings indicate that couples facing MS experience heightened levels of anxiety and depression. Additionally, couples who communicate more frequently about stress and are better at recognizing when their partner feels overwhelmed report better stress management and might even be effective in reducing the severity of anxiety and depression. Consequently, this could potentially enhance overall satisfaction within the relationship [8].

Employing a longitudinal design, Samios et al., [28] aimed to investigate how one partner’s adjustment at one point in time influences the other partner’s adjustment 12 months later. They found that one’s own adjustment at Time 1 predicted one’s adjustment at Time 2 for all outcomes, with significant partner effects observed for relationship satisfaction. Additionally, Özen et al., [23] noted that patients with MS had moderate levels of marital satisfaction, which was positively associated with perceived social support. 

Furthermore, in a secondary analysis of a randomised clinical trial, it was tested how improvements in partner support predict sexual satisfaction in a group of 81 participants with Multiple Sclerosis [2]. The findings revealed a significant association between perceived improvements in partner support and sexual satisfaction among MS patients. Notably, heightened levels of positive social support and reduced instances of negative partner support were both linked to enhanced sexual satisfaction. Consequently, these results underscore the nuanced impact of both positive and negative partner support on the sexual satisfaction outcomes for individuals living with MS.

Literature reviews also provide valuable insights into intimate relationships among individuals with MS. In their comprehensive review of 38 articles, Blundell Jones et al., [3] observed that MS not only induces physical changes in affected individuals but also impacts their self-perception and how they are perceived by others. The review highlighted the increasing need for care over time, which places significant demands on coping abilities and raises concerns about dependency. Emphasising the importance of accepting MS-related changes, the review suggested that such acceptance is crucial for escaping from detrimental relationship patterns and fostering effective coping strategies. However, the extent to which pre-diagnosis family/couple functioning styles influence interpersonal responses to MS remains unclear.

Further exploration reveals a multitude of factors associated with MS that detrimentally affect marital relationships, as elucidated by Delaney and Donovan [12]. These factors encompass challenges in adapting to the disease, difficulties with physical intimacy and sexual functions, communication impairments, caregiving-related stressors, anxiety, depression, and susceptibility to other health conditions.

Moreover, Uccelli’s [30] investigation into the impact of MS on family members, including romantic partners, unveiled a spectrum of emotions experienced by partners of individuals in the early stages of MS. Among these emotions were confusion, worry, anger, and shock. Thus, common themes included a sense of losing control over their lives and concerns about the worsening of the disease’s effects [30]. Partners of individuals with MS often experience emotional changes, including frustration, anger, and depression, which can impact the quality of the relationship. Uccelli, [30] also emphasized that studies on coping in couples with an MS-afflicted partner have identified specific characteristics and approaches associated with effective coping in this challenging situation. These include compatible communication and coping styles, viewing shifts in responsibilities as opportunities to deepen the relationship, and maintaining social roles and identity. Moreover, couples who adopt a present-focused mindset, seeking to minimize the influence of MS on their lives by accepting its presence and adjusting their expectations accordingly, find that this adaptive approach allows them to continue living meaningfully despite the challenges posed by the disease [30]. Finally, mutual respect, admiration, commitment, and a sense of obligation to the relationship and to each other appear to strengthen couples in facing the challenges posed by MS [30].

A powerful tool for investigating complex psychological and interpersonal phenomena, such as relationship trust, is network analysis (NA). In NA, each measured variable (e.g., a questionnaire item) is represented as a node, while the statistical relationships between variables (e.g., correlations or regression coefficients) are depicted as edges. This structure highlights the most central or influential nodes within a network, providing an intuitive framework for identifying critical intervention targets in research and clinical practice [7].

In the context of multiple sclerosis (MS), relationship trust is influenced by interconnected factors such as emotional support, decision-making, and perceived partner availability—complex relationships that traditional methods might overlook. Integrating a network approach can provide deeper insights into these interdependencies. Adopting this perspective responds to the growing need for integrative methodologies in behavioral neurology and lays the groundwork for future studies combining network analysis with dynamic, real-time data collection methods [27, 34, 35].

Understanding the dynamics of intimate relationships among individuals living with Multiple Sclerosis (MS) is crucial for addressing the unique challenges they face. Overall, the existing literature underscores the intricate relationship between MS and intimate relationships, emphasising the importance of support, communication, and adaptation in sustaining relationship satisfaction amidst the adversities associated with this condition.

Despite this acknowledgment, there remains a notable lack of studies regarding the specific factors that influence the quality of intimate relationships in individuals affected by MS. Thus, to address this gap, the current research aims to explore the multifaceted nature of intimate relationships in the context of MS. Specifically, the study adopts a network analysis approach, serving as an exploratory method, to further investigate the main factors that underlie the quality of relationships among patients with MS. Through a comprehensive examination of these interconnected variables, we aim to provide valuable insights that can inform interventions, support services, and ultimately improve the well-being of individuals and couples affected by MS. Considering the research thus far, to our knowledge, no explicit network analysis study has explored the key processes that underlie the quality of relationships within this group of individuals. Given the exploratory nature of network analysis, which maps the structure of a construct in the absence of strong prior theory rather than testing formal directional hypotheses [5], the present study aimed to identify the components of relationship trust that occupy central positions within the network structure and to examine how these components are interconnected in the lived experience of individuals diagnosed with multiple sclerosis. All findings should therefore be considered exploratory and preliminary in nature.

## Methods

### Participants

The study sample consisted of 189 individuals diagnosed with multiple sclerosis and hospitalized at the Neurology Hospital between January and December 2022. The inclusion criteria were as follows: (1) consent to participate in the study, (2) a minimum age of 18 years, and (3) a diagnosis of multiple sclerosis confirmed by a neurologist. The sample’s demographic composition included 123 females (65.1%), 61 males (32.3%), and five individuals who did not disclose their gender. Participants ranged in age from 19 to 92 years, with an average age of 43.39 years (SD = 12.86).

In terms of marital status, most participants were married (101 or 53.4%), followed by those who were divorced (45 or 23.8%), in partnerships (19 or 10.1%), single (21 or 11.1%), and widowed (2 or 1.1%). In terms of educational level, 22 (11.6%) individuals had completed secondary education, 55 (29.1%) had high school diplomas, 29 (15.3%) had post-secondary diplomas, 46 (24.3%) had undergraduate degrees, 29 (15.3%) had master’s degrees, and 4 (2.1%) held doctorates. Concerning residency, a significant portion of the sample lived in urban areas (134 or 70.9%), while 46 (24.3%) resided in rural areas. When asked about social support, 89 participants (47.1%) reported having social support, whereas 82 (43.4%) reported a lack of it. Additionally, most of the sample (168 or 88.9%) reported living with others, with a smaller segment (16 or 8.5%) living alone. Concerning the onset of the disease, participants reported various durations since diagnosis: 20 individuals (10.6%) indicated it was one year ago, 33 (17.5%) two years ago, 25 (13.2%) three years ago, 36 (19%) four years ago, and the largest group, 73 (38.6%), stated it was five years ago. Percentages may not sum to 100% because of missing responses on some demographic variables.

### Measures

Relationship Trust Scale. This measure assesses intimate relationships between partners, and it was used in the current study to investigate the relationship dynamics in a sample of individuals diagnosed with multiple sclerosis. Twenty items were derived from the work of Gottman and Silver [19] on the seven principles for making marriage work, adapted to assess the impact of a multiple sclerosis diagnosis on relationships. These items cover a range of dimensions, including communication, sexual intimacy, trust, shared interests, support, respect, fidelity, openness, and emotional safety. Item selection was guided by the theoretical framework of the Sound Relationship House [19], with inclusion criteria based on the relevance of each item to the construct of relationship trust as conceptualized within this framework. Specifically, items were selected if they directly assessed trust-relevant relational behaviors as defined by Gottman and Silver [19], and excluded if they referred to broader relationship satisfaction or personality traits unrelated to trust per se. The dichotomous (true/false) response format was not an arbitrary methodological decision, but was adopted directly from Gottman and Silver’s [19] original instruments, which consistently employ binary response options across multiple relationship assessment tools included in the book (e.g., the Shared Values Questionnaire), reflecting the theoretical assumption that certain relational behaviors and perceptions can be meaningfully captured as present or absent in a given relationship context. The use of binary data is also methodologically supported within the psychometric network analysis framework: for binary data, the Ising model represents a well-established estimation approach in which edges are parameterized as log-linear relationships, and mixed graphical models constitute the most general approach permitting categorical variables [5]. Respondents are encouraged to reflect on these aspects through true/false questions, providing quantitative insights into the relationship dynamics in individuals diagnosed with multiple sclerosis. Because the Relationship Trust Scale refers to a romantic partner, participants were instructed to answer the items with reference to their current partner or, if they were not currently in a relationship, to the most recent significant romantic relationship. Therefore, marital status categories such as single, divorced, or widowed do not necessarily indicate absence of a relationship referent for the RTS items.This scale showed excellent psychometric properties in the current sample (𝛼 = 0.90). We note, however, that the scale spans several content domains (e.g., communication, sexual satisfaction, fidelity, decision-making, and social support), and that internal consistency alone does not establish construct validity. In the network framework, this multidimensionality is not treated as a latent unidimensional structure but is instead modeled as a set of distinct yet interconnected components [4]. We nonetheless acknowledge the absence of formal exploratory or confirmatory factor analysis as a psychometric limitation, and the present study is explicitly positioned as a first exploratory mapping of this construct in MS populations, with psychometric refinement of the scale identified as a necessary next step before confirmatory approaches are applied [5]. See *Supplementary Table 1* for the items used in this study in the *Supplementary Materials* (*Supplementary Tables 1- Relationship Trust Scale*,* adapted after* [19]*).*

### Data collection and procedure

The current study employs a cross-sectional design, where participants completed the measurements in a single session. Recruitment occurred at the Neurology Hospital from January to December 2022. At the beginning of the session, participants provided informed consent, and all assessments were conducted using the conventional pencil and paper method.

### Data analysis plan

All statistical analyses were carried out using JASP version 0.18.3 [21] and RStudio version 2023.06.1 + 524 statistical software [9]. We utilized the “bootnet” [14] R statistical library for estimating/bootstrapping the network and running accuracy/stability testing and the “qgraph” [15] R statistical library for viewing the network nodes and connections between nodes.

In the current study, all networks were estimated via the Ising model [20]. In this model, research variables are represented as nodes, and the potential relationships between nodes are represented as edges. In order to derive the network structure, we employed the IsingFit network estimation procedure [10, 16, 24, 31], a method based on a combination between L1-regularized logistic regression (i.e., lasso regression) and model selection based on the Extended Bayesian Information Criterion (EBIC). The regularization is dependent on the EBIC parameter value (i.e., the hyperparameter gamma) which, in this study, was set to 0.25. This approach is the most appropriate to use when the network contains nodes that are characterised by discrete (dichotomous) variables.

The centrality indices and bootstrapped difference test were used to determine the relative importance of the network’s nodes (i.e., the betweenness, closeness, strength, and expected influence indices). Betweenness measures how important a node is on the average path between two other nodes, closeness measures how well a node is indirectly connected to other nodes, and strength measures how well a node is directly connected to other nodes [14]. Finally, expected influence measures the sum of all edge weights emanating from a given node while taking into account the edge’s positive or negative sign [25]. Expected influence is the best estimator in a network with both positive and negative edges because other centrality indices do not consider this polarity [25]. A total of 3000 bootstraps were used in estimating the potential statistically significant differences between centrality indices for nodes, edge weights, and to investigate the stability of the network centrality indices and the accuracy of network edges. See *Supplementary Material* for the *R Code used for estimating the network in the current study*.

## Results

### Network description

**Fig. 1:**
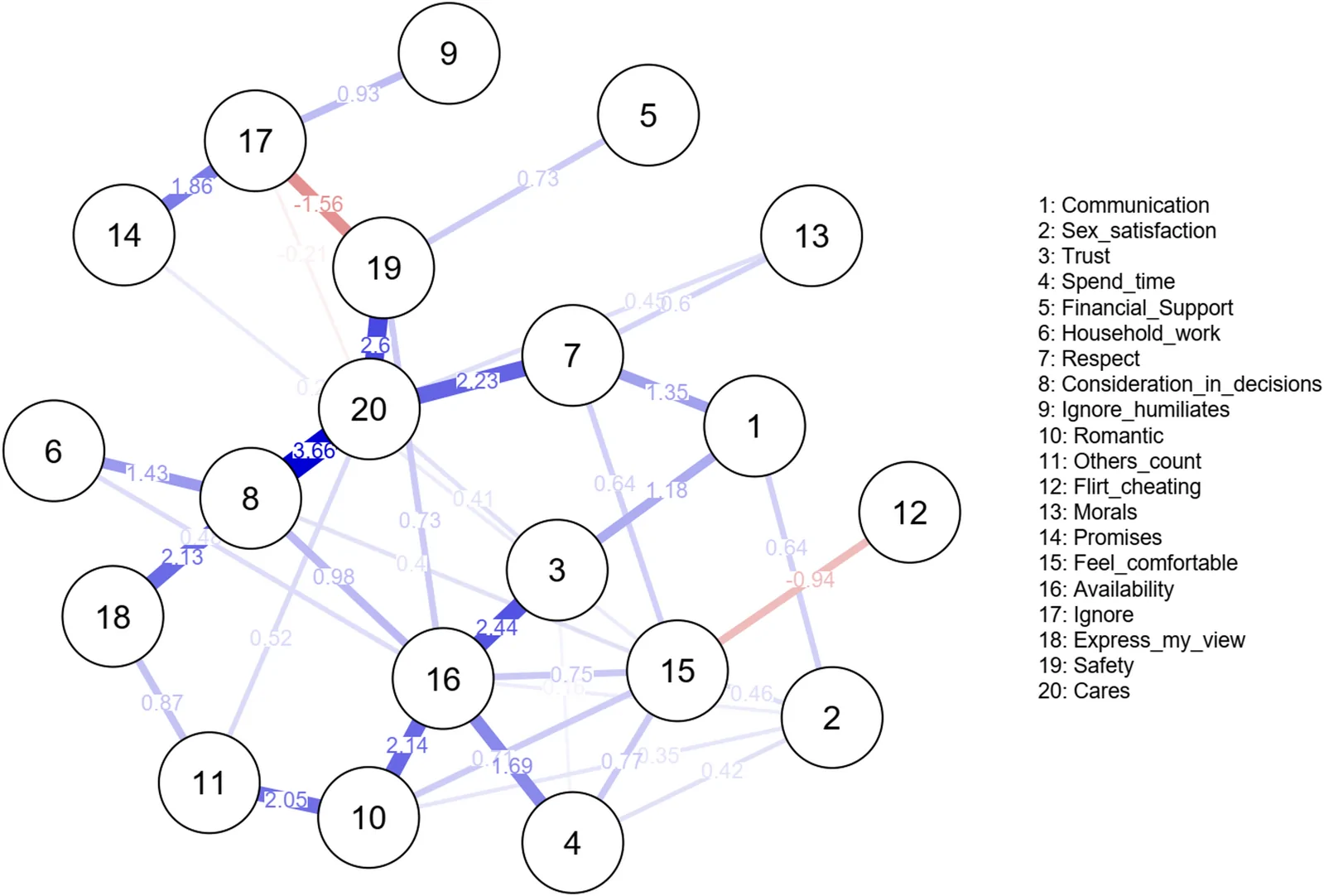
The network plot

The network plot (Fig. 1) indicated that the network was relatively sparse, with a sparsity coefficient of 0.805. The network consisted of 20 nodes and 37 non-zero edges (out of a maximum of 190 possible edges). The initial analysis indicated that the strongest regression coefficients in the network were those between Cares and Consideration in Decisions (ꞵ = 3.66), Cares and Safety (ꞵ = 2.60), Cares and Respect (ꞵ = 2.23), Availability and Trust (ꞵ = 2.44), Availability and Romantic (ꞵ = 2.14), or Availability and Spend Time (ꞵ = 1.69). While the majority of edges in the network were positive, a small number of negative edges were also observed, the strongest of which was between Ignore and Safety (β = −1.56). For a full detailed view of the regression coefficients between the nodes, see Table 1.

### Network analysis

**Fig. 2:**
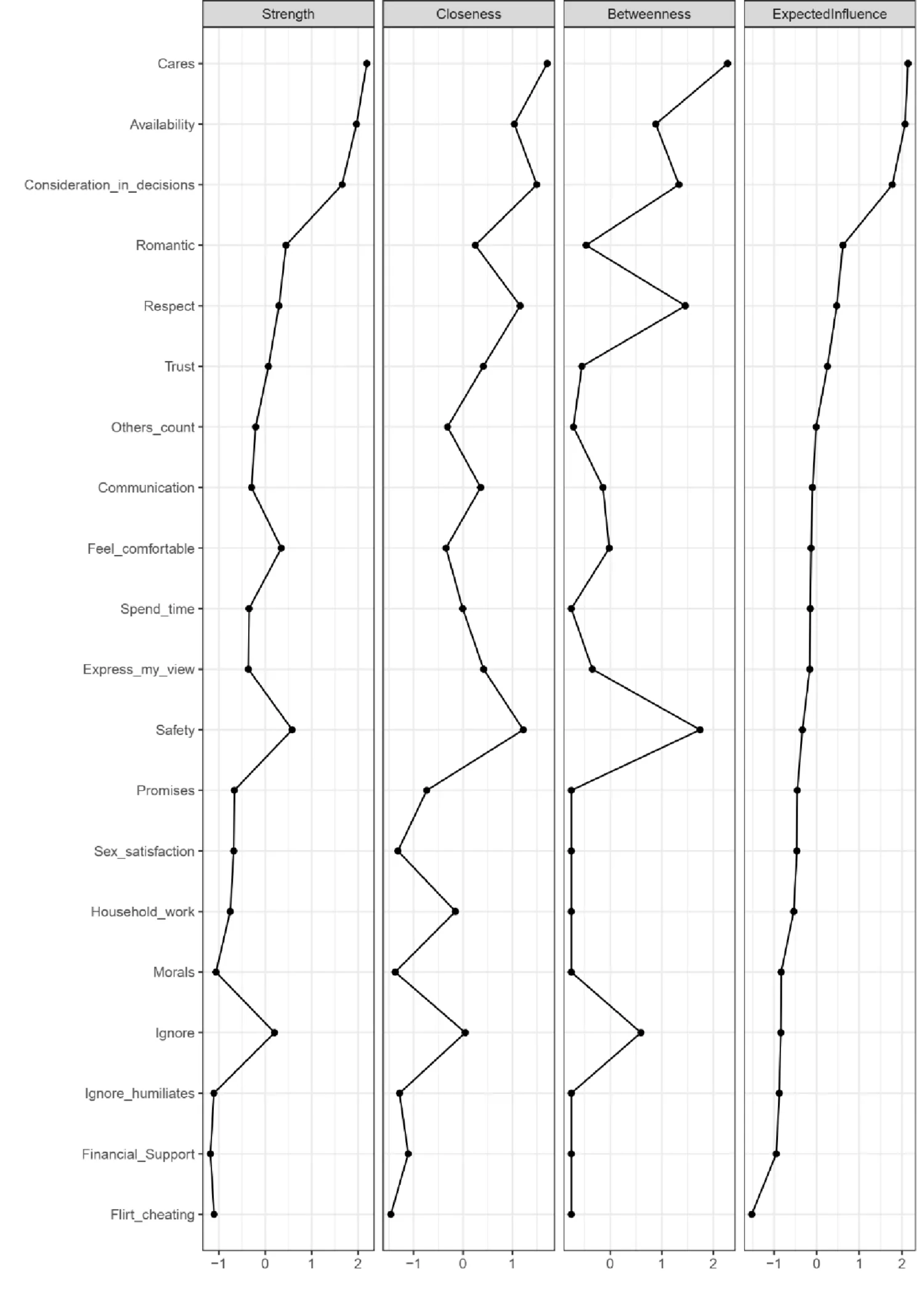
Centrality indices

Regarding the main analysis, the network is visually described in Fig. 1, while the centrality indices are plotted in Fig. 2 and described in Table 2. Firstly, with regard to betweenness, the Cares, Consideration in Decisions, Respect and Safety nodes had the highest z-scores, meaning that these nodes occurred most often on the paths between other nodes. Secondly, with regard to closeness, the Cares and Consideration in Decisions had the highest z-scores, meaning that these nodes were the closest to other nodes in the network. Thirdly, with regard to strength, the Cares, Availability and Consideration in Decisions had the highest z-scores, meaning that these nodes had the most and strongest connections to other nodes. Finally, in terms of expected influence, similarly, the Cares, Availability and Consideration in Decisions had the highest z-scores, meaning that these nodes were deemed to be the most influential in the network.

**Fig. 3:**
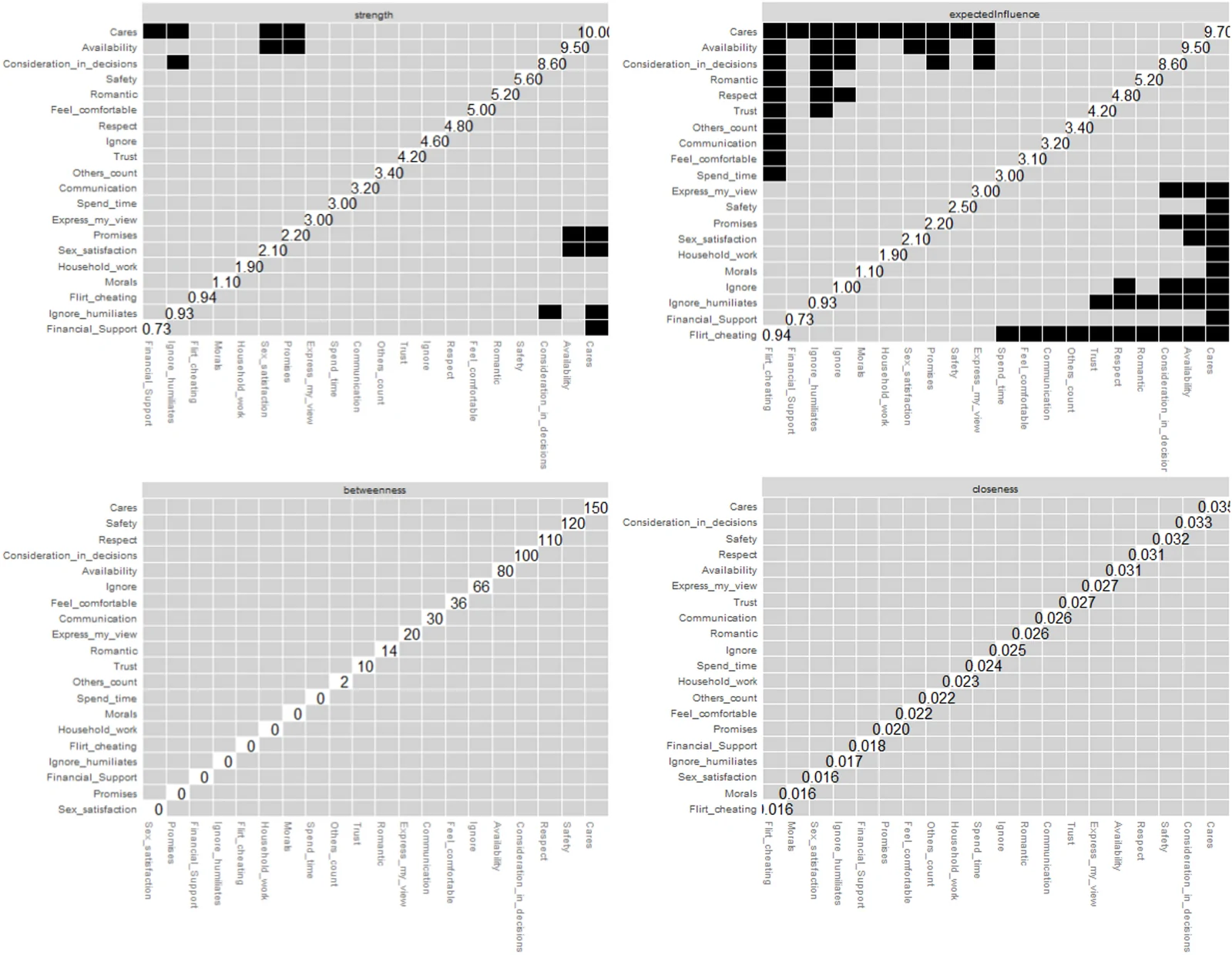
Statistically significant differences between the network nodes, with regard to centrality

The statistically significant differences between nodes, in terms of centrality indices, are presented in Fig. 3. For betweenness and closeness, there were no statistically significant differences between nodes. In terms of strength, the network analysis indicated that the Cares node had the highest strength (significantly higher than 21% of other nodes). In terms of expected influence, the network analysis indicated that the Cares node had the highest expected influence (significantly higher than 52.60% of other nodes), followed by the Availability (significantly higher than 31.57% of other nodes) and Consideration in Decisions (significantly higher than 26.31% of other nodes) nodes.

**Fig. 4:**
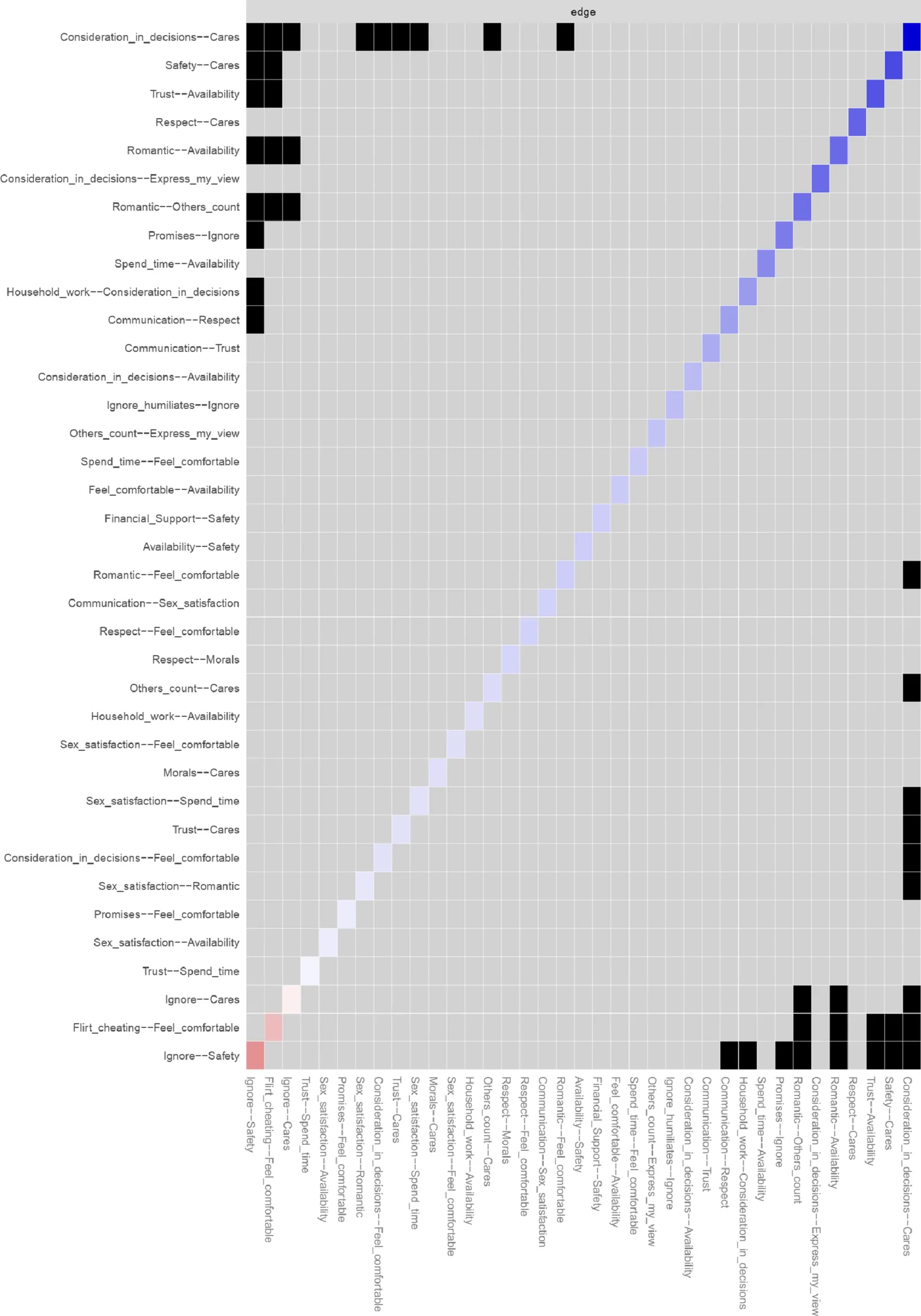
Edge differences

The statistically significant differences between network edges are presented in Fig. 4. The strongest edges were those between Cares and Consideration in Decisions (significantly stronger than 25% of other edges), followed by the edges between Romantic and Availability, or between Romantic and Others Count (both significantly stronger than 8.33% of other edges).

These results should be interpreted cautiously, given the fact that the network stability analysis indicated that the stability coefficients (CS-coefficient for betweenness = 0.048, closeness = 0.000, edge weight = 0.206, strength = 0.206, and expected influence = 0.286), for each centrality index, as well as for edges, did not reach the recommended threshold of 0.50 [14]. It should be noted, however, that as emphasized by Epskamp et al., [14], these cutoff values emerge from simulation studies and are somewhat arbitrary and should not be treated as definitive guidelines. In light of these values, and consistent with the recommendations of Bringmann et al., [6] that betweenness and closeness centrality may be especially unsuitable as measures of node importance in psychological networks due to violations of node distinctiveness and exchangeability assumptions, interpretations were refocused primarily on expected influence (CS-coefficient = 0.286), which exceeded the minimum recommended threshold, while betweenness (CS-coefficient = 0.048) and closeness (CS-coefficient = 0.000) were not interpreted, and strength-based findings (CS-coefficient = 0.206) were treated as exploratory only. Accordingly, all centrality-related findings reported here should be considered exploratory and preliminary.

**Table 1:** Regression indices for the main network analysis

Variable	Comm.	Sex sat.	Trust	Spend time	Financial Support	Household work	Respect	Cons. in decisions	Ignore humiliates	Rom.	Others count	Flirt cheat	Morals	Promises	Feel comf	Availability	Ignore	Express my view	Safety	Cares
Comm.	-																			
Sex sat.	0.64	-																		
Trust	1.19	0.00	-																	
Spend time	0.00	0.42	0.16	-																
Financial Support	0.00	0.00	0.00	0.00	-															
Household work	0.00	0.00	0.00	0.00	0.00	-														
Respect	1.35	0.00	0.00	0.00	0.00	0.00	-													
Cons. in decisions	0.00	0.00	0.00	0.00	0.00	1.43	0.00	-												
Ignore humiliates	0.00	0.00	0.00	0.00	0.00	0.00	0.00	0.00	-											
Rom.	0.00	0.35	0.00	0.00	0.00	0.00	0.00	0.00	0.00	-										
Others count	0.00	0.00	0.00	0.00	0.00	0.00	0.00	0.00	0.00	2.05	-									
Flirt cheat	0.00	0.00	0.00	0.00	0.00	0.00	0.00	0.00	0.00	0.00	0.00	-								
Morals	0.00	0.00	0.00	0.00	0.00	0.00	0.60	0.00	0.00	0.00	0.00	0.00	-							
Promises	0.00	0.00	0.00	0.00	0.00	0.00	0.00	0.00	0.00	0.00	0.00	0.00	0.00	-						
Feel comf.	0.00	0.46	0.00	0.77	0.00	0.00	0.64	0.40	0.00	0.71	-0.94	0.00	0.00	0.29	-					
Availability	0.00	0.25	2.44	1.69	0.00	0.48	0.00	0.98	0.00	2.14	0.00	0.00	0.00	0.00	0.75	-				
Ignore	0.00	0.00	0.00	0.00	0.00	0.00	0.00	0.00	0.93	0.00	0.00	0.00	0.00	1.86	0.00	0.00	-			
Express my view	0.00	0.00	0.00	0.00	0.00	0.00	2.13	0.00	0.00	0.87	0.00	0.00	0.00	0.00	0.00	0.00	0.00	-		
Safety	0.00	0.00	0.00	0.00	0.73	0.00	0.00	0.00	0.00	0.00	0.00	0.00	0.00	0.00	0.00	0.73	-1.56	0.00	-	
Cares	0.00	0.00	0.41	0.00	0.00	0.00	2.23	3.66	0.00	0.00	0.52	0.00	0.45	0.00	0.00	0.00	-0.21	0.00	2.60	-

Comm. = Communication; Sex sat. = Sex satisfaction; Cons. in decisions = Consideration in decisions; Rom = romantic; Feel comf. = feel comfortable

**Table 2:** Centrality measures

Centrality measures per variable				
Variable	Betweenness	Closeness	Strength	Expected influence
Communication	-0.14	0.36	-0.30	-0.10
Sex_satisfaction	-0.76	-1.32	-0.68	-0.46
Trust	-0.55	0.41	0.07	0.26
Spend_time	-0.76	-0.01	-0.35	-0.15
Financial_Support	-0.76	-1.11	-1.18	-0.95
Household_work	-0.76	-0.15	-0.75	-0.54
Respect	1.46	1.16	0.30	0.47
Consideration_in_decisions	1.33	1.49	1.66	1.78
Ignore_humiliates	-0.76	-1.29	-1.11	-0.87
Romantic	-0.47	0.25	0.45	0.62
Others_count	-0.72	-0.31	-0.21	-0.01
Flirt_cheating	-0.76	-1.46	-1.10	-1.52
Morals	-0.76	-1.37	-1.06	-0.83
Promises	-0.76	-0.73	-0.67	-0.45
Feel_comfortable	-0.02	-0.34	0.35	-0.13
Availability	0.88	1.04	1.97	2.08
Ignore	0.60	0.05	0.20	-0.84
Express_my_view	-0.35	0.42	-0.36	-0.16
Safety	1.74	1.22	0.58	-0.33
Cares	2.28	1.70	2.19	2.14

## Discussion

The purpose of the current study was to estimate the network of the Relationship Trust Scale among individuals diagnosed with MS and identify the most central nodes. Current results suggested that, in terms of expected influence, Cares, Availability, and Consideration in Decisions appeared to occupy relatively central positions in the network. Importantly, however, these findings should be interpreted as exploratory and preliminary, given that the stability coefficients fell below recommended thresholds and that centrality rankings may therefore not be replicable across different samples [14]. Interpretation is consequently restricted primarily to expected influence (CS-coefficient = 0.286), which exceeded the minimum recommended threshold, whereas betweenness and closeness were not interpreted and strength-based findings were treated as exploratory only [6]. With these caveats in mind, the pattern of findings may underscore the significance of emotional care and support (Item: "My partner cares about my worries, feelings, joys, and troubles.“), availability during emotional distress (Item: “Whenever I am upset my partner is always available, supportive, and I feel like he/she’s there for me.“), and consideration in decision-making (Item: “My partner takes me into account when making a decision.“) in the relationships of individuals diagnosed with MS.

These results align with previous research indicating that emotional care (e.g., showing interest in the experiences and emotional well-being of one’s partner – cognitive aspect) and availability of support (e.g., being present to offer support during emotional periods – behavioral aspect) are highly important for good relationship quality among individuals with MS [2]. This is particularly crucial given that individuals diagnosed with MS often experience elevated levels of stress, depression, and anxiety, thus requiring effective coping mechanisms [8]. Additionally, the required support extends not only to the emotional level but also to the decision-making process, as this process is highly relevant for these individuals. Consideration in decision-making might be particularly relevant due to the difficulties faced by individuals with multiple sclerosis, including emotional and functional challenges (e.g., motor weakness, coordination difficulties, cognitive impairment), and the array of decisions they encounter, such as those related to medical matters, domestic responsibilities, and financial considerations [29].

Further, the network was characterized predominantly by positive associations, with the strongest connections observed between Cares and Consideration in Decisions, Cares and Safety, and Cares and Respect. These positive edges suggest that perceptions of being cared for, respected, and included in decisions tend to co-occur within the relational experience of individuals with MS, forming a tightly interconnected cluster around emotional care. Alongside these positive associations, the network also contained a smaller number of negative edges. The strongest of these was the negative association between Ignore and Safety (β = −1.56), which suggests that higher levels of perceived partner ignorance may be inversely related to feelings of safety within the relationship. Although this pattern would be consistent with Gottman and Silver’s (2015) conceptualization of emotional distance as a form of nonsexual betrayal that undermines relational safety, we interpret it with considerable caution, as the stability of edge-level estimates in the present network was limited (CS-coefficient for edge weights = 0.206) and the directionality of such associations cannot be established within a cross-sectional, undirected network. Future studies employing larger samples and Directed Acyclic Graph (DAG) approaches could formally test whether perceived partner ignorance precedes diminished feelings of safety [5].

From a practical standpoint, the present exploratory findings may offer tentative directions for psychosocial interventions aimed at enhancing partners’ capacity to provide emotional support and care to individuals with MS, particularly during periods of anxiety or sadness. Furthermore, fostering availability to assist individuals with MS in regulating their negative emotions may enhance the overall quality of couple relationships. Additionally, it is important to empower partners of individuals with multiple sclerosis to effectively include them in the decision-making process, even though difficulties might arise in this process. Thus, our findings point towards some potential practical implications for prevention and intervention programs tailored for individuals diagnosed with MS. Specifically, psychosocial interventions, such as cognitive-behavioral interventions, could prioritize these three relationship processes identified within the structure of the RTS network, namely Care, Availability, and Consideration in decisions. Utilizing network analysis, which enables the identification of intervention points based on edges or nodes [7], the current results could inform interventions targeting relationship issues in individuals diagnosed with MS, thereby laying the groundwork for network-informed interventions [26].

The current study also has several important limitations. Firstly, a significant limitation pertains to the nature of the data collected. Specifically, we relied solely on self-report measures, raising concerns about potential bias in the data. Utilizing ecological momentary assessments and gathering data from multiple informants (e.g., partners, caregivers) could further bolster our conclusions regarding the most relevant relationship processes in individuals diagnosed with MS. Secondly, the current sample predominantly comprised female participants, potentially limiting the generalizability of the results. Future studies should strive for a more balanced gender representation to ensure the findings are applicable across diverse demographic groups. A further limitation concerns the clinical heterogeneity of the sample. Participants were treated as a single group, and the analysis did not account for clinical variables such as disease subtype, disability level as measured by the Expanded Disability Status Scale, or clinical severity, all of which may influence psychosocial functioning and relationship dynamics in distinct ways. The present analysis therefore does not allow conclusions about how the network structure of relationship trust might differ across clinical profiles. While treating the full sample as a single group is consistent with the exploratory aim of mapping this construct for the first time and with standard practice in initial network studies [5], future studies should include clinical variables such as disease subtype and Expanded Disability Status Scale scores and employ larger samples to enable network comparison analyses across distinct clinical profiles, for instance using the Network Comparison Test [32].

A related and central limitation concerns sample size and the resulting low network stability. To examine this more rigorously, we conducted a post-hoc power simulation using the powerly package [11], a Monte Carlo method designed for sample size analysis in the context of network models. Using the empirical network density (density = 0.20), 20 nodes, a sensitivity target of 0.80, and a power threshold of 0.80, the simulation indicated that approximately 479 participants (95% CI: 467–495) would be required to achieve adequate statistical power. As powerly’s validated implementation currently covers the Gaussian graphical model, this estimate employed a Gaussian graphical model approximation; the recommended sample size should therefore be interpreted as an approximation rather than an exact estimate. The current sample falls below this threshold, which is consistent with the low stability coefficients observed and further supports interpreting all findings as exploratory and preliminary. Recruiting samples of this size in clinical multiple sclerosis populations is inherently challenging given the relatively low prevalence of the condition and the need for specialized clinical recruitment, a constraint common to network-analytic studies in clinical populations [5]. We strongly recommend that future studies prioritize larger samples to enable more stable and reliable network estimation.

Thirdly, a significant limitation is associated with the cross-sectional design of this study. While cross-sectional data provide valuable insights, they do not permit causal inferences. Our results suggest that care, availability, and consideration in decision-making are potential primary candidates for testing in longitudinal or experimental studies. Additionally, it remains unclear whether the patterns identified at the group level are applicable at an individual level, and the cross-sectional design cannot capture the relevance of these relationship processes over time. Future studies could employ time-series analysis and collect data on multiple occasions (e.g., intensive longitudinal designs) to better understand the complex dynamics of relationship processes over time in individuals diagnosed with MS.

Despite these limitations, the current study offers valuable preliminary insights into candidate relationship processes within the RTS network that may warrant further investigation in larger, more adequately powered samples. As hypothesis-generating findings, they tentatively highlight the significance of emotional care and support, the importance of partner availability during emotional distress, and the inclusion of individuals in the decision-making process within the relationships of those diagnosed with MS.

In conclusion, this study provides an exploratory first mapping that suggests a potentially relevant role for these three processes within the intimate relationships of individuals diagnosed with MS, further enriching our understanding of key dynamics in this domain. Consequently, interventions targeting these processes in the intimate relationships of these individuals show promise for enhancing relationship quality and well-being, and promoting a more adaptive response to this specific adversity.

## Supplementary Information

Below is the link to the electronic supplementary material.

Supplementary Material 1

## Data Availability

The data that support the findings of this study are available from the corresponding author, upon request.
