# Supplementary material for: Relationship trust in individuals with multiple sclerosis: a network analysis approach
Source: Acta Neurol Belg. 2026 Jul 8;126(5):1699–711. doi: 10.1007/s13760-026-03130-9 (PMC13713998; doi:10.1007/s13760-026-03130-9)
Supplement: Supplementary file 1 [file 13760_2026_3130_MOESM1_ESM.docx]

**Table S1. Relationship Trust Scale, adapted after Gottman and Silver, 2015**

**ITEM 1. COMMUNICATION**

We communicate the same way we did before the onset of my illness.

TRUE ☐	FALSE ☐

**ITEM 2. SEX SATISFACTION**

I am as satisfied as I was before the onset of my illness regarding the sexual relationship.

TRUE ☐	FALSE ☐

**ITEM 3. TRUST**

I trust my partner as much as I did before the onset of my illness.

TRUE ☐	FALSE ☐

**ITEM 4. SPEND TIME**

We spend time together the same way we did before the illness. We do the same activities and have the same hobbies as before.

TRUE ☐	FALSE ☐

**ITEM 5. FINANCIAL SUPPORT**

My partner supports me financially.

TRUE ☐	FALSE ☐

**ITEM 6. HOUSEHOLD WORK**

My partner helps around the house with different household chores.

TRUE ☐	FALSE ☐

**ITEM 7. RESPECT**

My partner respects me.

TRUE ☐	FALSE ☐

**ITEM 8. CONSIDERATION IN DECISION**

My partner takes me into account when making a decision.

TRUE ☐	FALSE ☐

**ITEM 9. IGNORE/HUMILIATES**

My partner ignores or humiliates me.

TRUE ☐	FALSE ☐

**ITEM 10. ROMANTIC**

My partner is romantic towards me.

TRUE ☐	FALSE ☐

**ITEM 11. OTHERS/COUNT**

My partner shows others how much I mean to him/her.

TRUE ☐	FALSE ☐

**ITEM 12. FLIRT/CHEATING**

My partner flirts with other people/I fear he/she might cheat on me.

TRUE ☐	FALSE ☐

**ITEM 13. MORALS**

My partner is a moral person.

TRUE ☐	FALSE ☐

**ITEM 14. PROMISES**

My partner does not keep his/her promises.

TRUE ☐	FALSE ☐

**ITEM 15. FEEL COMFORTABLE**

I feel comfortable sharing anything with my partner.

TRUE ☐	FALSE ☐

**ITEM 16. AVAILABILITY**

Whenever I am upset my partner is always available, supportive, and I feel like he/she’s there for me.

TRUE ☐	FALSE ☐

**ITEM 17. IGNORE**

My partner ignores me.

TRUE ☐	FALSE ☐

**ITEM 18. EXPRESS MY VIEW**

I am not afraid to share my opinion if we argue.

TRUE ☐	FALSE ☐

**ITEM 19. SAFETY**

I feel safe in this relationship.

TRUE ☐	FALSE ☐

**ITEM 20. CARES**

My partner cares about my worries, feelings, joys, and troubles.

TRUE ☐	FALSE ☐

**#R script for the analyses**

library("bootnet")

Data <- read.csv("INPUT PATH TO DATABASE", header = TRUE)

Network <- estimateNetwork(Data, default = "IsingFit", tuning = 0.25,

missing = "listwise", verbose = TRUE, rule = "OR", split = "mean")

plot(Network, layout = "spring", labels = TRUE)

library("qgraph")

centralityPlot(Network, include = c("Strength", "Closeness", "Betweenness", "ExpectedInfluence"),

scale = "z-scores", orderBy = "ExpectedInfluence")

boot1 <- bootnet(Network, nBoots = 3000, nCores = 8, statistics = c("edge", "strength", "closeness", "betweenness", "expectedInfluence"))

plot(boot1, labels = TRUE, order = "sample")

boot2 <- bootnet(Network, nBoots = 3000, type = "case", nCores = 8, statistics = c("edge", "strength", "closeness", "betweenness", "expectedInfluence"))

plot(boot2)

corStability(boot2, statistics = "all")

plot(boot1, "edge", plot = "difference", onlyNonZero = TRUE, order = "sample")

plot(boot1, "betweenness", order = "sample")

plot(boot1, "closeness", order = "sample")

plot(boot1, "strength", order = "sample")

plot(boot1, "expectedInfluence", order = "sample")
